# Culture-Independent Genotyping, Virulence and Antimicrobial Resistance Gene Identification of *Staphylococcus aureus* from Orthopaedic Implant-Associated Infections

**DOI:** 10.3390/microorganisms9040707

**Published:** 2021-03-30

**Authors:** J. Christopher Noone, Fabienne Antunes Ferreira, Hege Vangstein Aamot

**Affiliations:** 1 Department of Microbiology and Infection Control, Akershus University Hospital, 1478 Lørenskog, Norway; fabienne.ferreira@ufsc.br (F.A.F.); Hege.Vangstein.Aamot@ahus.no (H.V.A.); 2 Faculty of Medicine, University of Oslo, 0316 Oslo, Norway; 3 Departamento de Microbiologia, Imunologia e Parasitologia, Universidade Federal de Santa Catarina (UFSC), Florianópolis 88040-900, Brazil; 4 Department of Clinical Molecular Biology (EpiGen), Akershus University Hospital and University of Oslo, 1478 Lørenskog, Norway

**Keywords:** nanopore sequencing, shotgun metagenomics, culture-independent, *Staphylococcus aureus*, genotyping, virulence genes, antimicrobial resistance, orthopaedic implant-associated infections

## Abstract

Our culture-independent nanopore shotgun metagenomic sequencing protocol on biopsies has the potential for same-day diagnostics of orthopaedic implant-associated infections (OIAI). As OIAI are frequently caused by *Staphylococcus aureus*, we included *S. aureus* genotyping and virulence gene detection to exploit the protocol to its fullest. The aim was to evaluate *S. aureus* genotyping, virulence and antimicrobial resistance genes detection using the shotgun metagenomic sequencing protocol. This proof of concept study included six patients with *S. aureus*-associated OIAI at Akershus University Hospital, Norway. Five tissue biopsies from each patient were divided in two: (1) conventional microbiological diagnostics and genotyping, and whole genome sequencing (WGS) of *S. aureus* isolates; (2) shotgun metagenomic sequencing of DNA from the biopsies. Consensus sequences were analysed using spaTyper, MLST, VirulenceFinder, and ResFinder from the Center for Genomic Epidemiology (CGE). MLST was also compared using krocus. All *spa*-types, one CGE and four krocus MLST results matched Sanger sequencing results. Virulence gene detection matched between WGS and shotgun metagenomic sequencing. ResFinder results corresponded to resistance phenotype. *S. aureus spa*-typing, and identification of virulence and antimicrobial resistance genes are possible using our shotgun metagenomics protocol. MLST requires further optimization. The protocol has potential application to other species and infection types.

## 1. Introduction

The possible ramifications of orthopaedic implant-associated infections (OIAI) are salient, including reduced function, poorer treatment outcome, and increased mortality. Delayed or poorly targeted treatment can lead to selection for antimicrobial resistant bacterial strains, revision surgery, or removal of the implant device [1,2]. The current gold standard for microbiological diagnostics of OIAI is culturing [3], which has been criticized for both its sensitivity and the time to diagnosis [4]. Culture-independent shotgun metagenomic sequencing looks to be a promising alternative in the effort to provide rapid, accurate diagnoses of OIAI.

Our recently tested protocol using nanopore shotgun metagenomic sequencing directly on tissue biopsies has the potential to facilitate same-day diagnostics and administration of targeted treatment [5]. As OIAI are most frequently caused by *Staphylococcus aureus* [6], we have included *S. aureus* protein A (*spa*)-typing, multilocus sequence typing (MLST), and virulence gene detection to exploit the new protocol to its fullest.

Recent advances in the challenging area of metagenomic bacterial genome assembly in the form of the specialized long-read metagenomic assembly program metaflye [7] and sequence polishing tools such as medaka (https://github.com/nanoporetech/medaka) have made the inclusion of these analyses relatively simple. Metaflye and medaka facilitate the rapid assembly and correction of the long, error-prone nanopore *S. aureus* reads obtained from the metagenomic sequencing of OIAI in earlier work [5].

*spa*-typing and MLST are used in the molecular characterization of *S. aureus*-OIAI [8]. Additionally, the two techniques have been employed to confirm the existence of commonly occurring *S. aureus* clones in OIAI distinct to geographically restricted population groups and to confirm the concordance to the patients’ own carrier clones, thus suggesting the route of infection [9].

The high frequency of *S. aureus* OIAI is, at least in part, due to the organism’s array of virulence factors, some of which are used to evade the host immune response [10]. The ability to distinguish between high and low cytotoxic *S. aureus* can steer therapeutic treatment and facilitate the clearance of potentially persistent *S. aureus* infections [11].

Finally, successful treatment of an *S. aureus* OIAI is contingent upon timely and appropriate antimicrobial treatment. Conventional phenotypic antimicrobial susceptibility testing (AST) is time consuming and has been criticized for its subjectivity, lack of reproducibility, and accuracy [12,13,14,15]. Rapid and reliable prediction of antimicrobial resistance (AMR) phenotype based on detection of AMR genes can potentially reduce time to targeted OIAI treatment. However, the simple detection of AMR gene presence can be ambiguous when trying to predict AMR phenotype [5]. Improvements to the web-based ResFinder (Center for Genomic Epidemiology, CGE; https://cge.cbs.dtu.dk/services/ResFinder/) have the potential to facilitate more reliable genome-based prediction AMR phenotype [16].

The aim of this work was to evaluate *S. aureus* genotyping, and virulence and antimicrobial resistance gene detection using the expanded nanopore shotgun metagenomic sequencing protocol directly from soft tissue biopsies, comparing the results to the respective current standard methods.

## 2. Materials and Methods

This prospective proof of concept study included acute OIAI patients undergoing first revision surgery at Akershus University Hospital, Norway. The patients are a sub-set of those included in an earlier study testing a culture-independent shotgun metagenomic sequencing protocol for rapid diagnostics of OIAI patients [5]. Patients with *S. aureus* OIAI were eligible. Five soft tissue biopsies were analysed from each patient.

**Figure 1:**
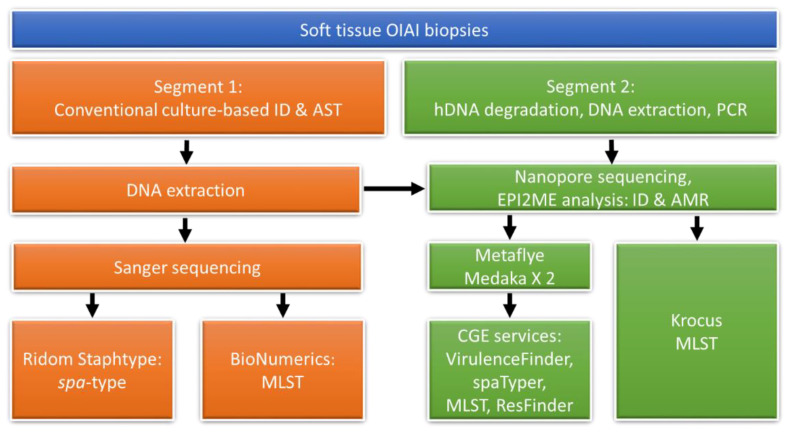
Flow chart of the parallel analyses of each orthopaedic implant associated infection (OIAI) patient’s two biopsy segments. Following conventional analysis of *S. aureus* isolates, extracted isolate DNA was analysed comparatively to DNA extracted from the respective patients’ biopsies. Additional abbreviations are as follows: AMR (antimicrobial resistance), AST (antimicrobial susceptibility testing), CGE (Center for Genomic Epidemiology), hDNA (human DNA), ID (identification), MLST (multilocus sequence typing), *spa* (*S. aureus* protein A gene).

Patient biopsies were taken from areas adjacent the implant. Each patient biopsy was divided into two segments. One segment was cultivated following conventional microbiological diagnostics including pathogen identification with MALDI-TOF (Matrix-Assisted Laser Desorption/Ionization-Time of Flight) mass spectrometry and antimicrobial susceptibility testing in accordance with EUCAST (European Committee on Antimicrobial Susceptibility Testing) guidelines, as described in earlier work [17]. The other segment was initially frozen in −80 °C awaiting DNA extraction and subsequent nanopore sequencing. An analysis flowchart is depicted in Figure 1.

### 2.1. DNA Extraction

The cultured *S. aureus* isolate DNA was extracted using PureLink Genomic DNA Mini Kit (Invitrogen, MA, USA), following the manufacturer’s protocol apart from the substitution of the lysozyme treatment by an incubation with 10 µL lysostaphin (5 mg/mL) (Sigma-Aldrich, St Louis; MO, USA) in 180 µL phosphate buffered saline (PBS) at 37 °C for 45 min.

DNA from the tissue biopsies was extracted using Ultra-Deep Microbiome Prep kit (Molzym, Bremen, Germany) optimized for enhanced degradation of human DNA (hDNA), as described in earlier work [18].

### 2.2. Spa-Typing and MLST

Sanger-based *spa*-typing and MLST typing of the isolates were performed as previously described [19,20]. Sequencing was performed using BigDye Terminator v1.1 Cycle Sequencing Kit and the 3130xl Genetic Analyzer (Applied Biosystems, Waltham, MA, USA). Ridom Staphtype software v.2.2.1 (Ridom GmbH, Münster, Germany) was used to assign *spa*-type. The BioNumerics v.7.6.3 MLST online database was used for assignment of sequence type.

### 2.3. Nanopore Sequencing

WGS of the *S. aureus* isolates was performed using rapid barcoding kit (SQK-RBK004, Oxford Nanopore Technologies; ONT, Oxford, UK). All six isolates were sequenced on the same flow cell. Input and reagent volumes were doubled. Otherwise, library prep and sequencing were carried out following the manufacturer’s protocol (RBK_9054_v2_revM_14Aug2019).

Shotgun metagenomic sequencing was performed on DNA extracted directly from patient biopsies using rapid PCR barcoding kit (SQK-RPB004) as described earlier [5].

All nanopore sequencing was performed using ONT’s GridION X5 Mk1 sequencing platform and R9.4.1 FLO-MIN 106 flow cells (ONT). Each sequencing run was set to a duration of 48 h.

### 2.4. Nanopore Sequencing Data Analysis

Raw sequencing data were analysed and basecalled using the graphical user interface MinKNOW 2.0 and Guppy basecaller v3.0.6 (ONT). ONT’s cloud-based EPI2ME workflows were used for quality control and demultiplexing (Barcode), species identification (WIMP), and AMR gene identification (ARMA). EPI2ME workflows were employed using default Q-score ≥ 7.

Both metagenomic and isolate reads were assembled with metaflye [7]. The assembled contigs were polished with two iterations of medaka (https://nanoporetech.github.io/medaka/). The assembled metagenomic genomes were then aligned to the assembled isolate genomes in Geneious Prime (v.2021.0.3) to assess the metagenomic breadth of coverage of each’s respective isolate.

The resulting medaka consensus sequences were further analysed using spaTyper 1.0, MLST 2.0, and VirulenceFinder 2.0 services from CGE (www.genomicepidemiology.org). ResFinder 4.1 was included (both acquired antimicrobial resistance genes and chromosomal point mutations models), as previously published EPI2ME AMR gene detection using the current metagenomic protocol was suboptimal [5]. Both VirulenceFinder and ResFinder were set to 90% identity threshold and 60% minimum length threshold.

Further comparison of MLST was carried out on the uncorrected isolate and metagenomic nanopore reads using krocus, a k-mer-based typing software, designed for typing from uncorrected long-read sequence data [21].

## 3. Results

### 3.1. Metaflye Assemblies

Based on previously published metagenomic sequencing data [5], metaflye assemblies containing less than approximately 33,000 *S. aureus* reads yielded insufficient depth of coverage for downstream analyses (median depth ≤ 11X). Accordingly, six of 17 eligible patients’ biopsies were analysed further. Their metagenomic sequencing runs ranged in *S. aureus* reads from 33,087 to 2,474,387.

Four patients’ biopsies (IDs 111, 114, 140, 141) were sequencing positive for other staphylococci: *S. lugdunensis* (IDs 111, 114, 140, 141), *S. epidermidis* (IDs 114, 140), and *S. argenteus* (IDs 114, 140). However, the amount of reads for these bacteria was well below 1% of the total bacterial reads, and no assemblies resulted. *S. aureus* was the only bacterium assembled into contigs by metaflye across the sample set. Two patients’ biopsies’ (IDs 139, 141) metaflye assemblies contained several hDNA contigs in addition to *S. aureus*. Detailed sequencing and assembly metrics of the included patients are detailed in the supplementary data, Tables S1 and S2. The *S. aureus* in these six samples were identified within an hour of sequencing start using the WIMP analysis carried out in earlier work [5]. Metaflye and the two iterations of Medaka took approximately from 10 min to two hours combined, relative to the number of reads.

The metagenomic assemblies’ median breadth of coverage of the isolates’ *S. aureus* chromosome was 95% [69.2%–99.9%]. Median pair-wise similarity for the metagenomic-isolate chromosome alignments was 99.88% [99.2%–99.96%]. Two isolates’ assemblies revealed plasmids: ID 111, breadth of coverage 50% and pair-wise similarity 99.95%; and ID 114, breadth of coverage 14.3% and pair-wise similarity 100% (Supplementary material, Figure S1).

### 3.2. Spa-Type

*spa*-typing results showed 100% agreement between metagenomic and Sanger sequencing. The following *spa*-types were detected (patient ID): t2413 (ID 111), t2439 (ID 114), t122 (ID 128), t276 (ID 139), t084 (ID 140), and t5221 (ID 141). Whole genome nanopore sequencing of the respective *S. aureus* isolates matched in all cases as well.

### 3.3. MLST

The MLST results are presented in Table 1. The metagenomic sequencing analysed with CGE resulted in one exact match with Sanger sequencing (ID 140). Raw metagenomic nanopore reads analysed with krocus matched unequivocally in four patients’ samples (IDs 111, 114, 139, 141). One patient’s samples (ID 140) were typed as ST6326 or ST582 due to a sequencing error in *arcC*. *arcC* is one of the seven housekeeping genes sequenced to determine *S. aureus* sequence type. The sequencing errors here entailed the basecalling calling of one adenine (A) too few in a homopolymeric A region. This led to 100% matches for both *arcC* alleles 655 (ST6326) and 13 (ST582), leading the typing error of some reads. Krocus failed to type one patient’s metagenomic sample (ID 128).

**Figure 2:**
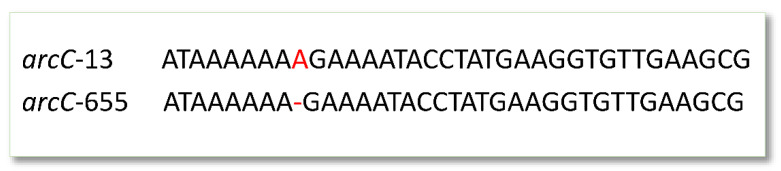
Example of the systematic nanopore sequencing homopolymeric A deletion. The deletion was present in all nanopore-sequenced *arcC* loci in this study, with the exception of ID 140. Shown here, is a portion of the nanopore sequenced *arcC* locus *S. aureus* isolate for patient 111 (identical to the metagenomic sequence) aligned with the Sanger sequenced isolate for the same patient. Both nanopore protocols resulted in 100% matches for *arcC* allele 655 due to the basecalling error. Krocus matched Sanger sequencing, identifying this patient’s *arcC* allele as allele 13.

The MLST allele sequence discrepancies between nanopore sequenced samples and Sanger sequencing were most often in the form of the aforementioned missing A in a homopolymeric A region towards the 3′-end of *arcC* (Figure 2).

Additional non-homopolymeric A deletion errors occurred in the nanopore sequenced isolate DNA from patients 111 and 140. These patients’ sample sequencing results had the following sequencing errors: a cytosine > thymine (C > T) substitution in *yqil* (ID 111); and a guanine (G) insert in *tpi*, a C > T substitution in *pta*, and a homopolymeric T insertion in *gmk* (ID 140).

### 3.4. Virulence Genes

VirulenceFinder results of the metagenomic protocol matched the isolate WGS results 100%. A complete list of all *S. aureus* virulence genes detected in the study is found in the supplemental data, Table S3.

### 3.5. Resistance

ResFinder AMR gene detection results of the metagenomic protocol predicted the observed culture-based AST resistance phenotypes in all cases. The phenotypes included penicillin (all samples) and tetracycline resistance (ID 128), based respectively on the detection of the genes *blaZ* and *tetK*.

The EPI2ME ARMA workflow detected the presence of genes which matched the respective AST resistance phenotypes in all cases; however, EPI2ME analysis also detected the presence of other putative AMR genes that were not observed in the respective AST phenotypes. The genes included *tet38*, *sav1866*, *mepA*, *mepR*, *mgrA*, *arlS*, *arlR*, and *vgaA* (Supplementary data, Table S4).

## 4. Discussion

This prospective proof of concept study demonstrates that, in addition to the previously reported rapid culture-free pathogen identification [5], *spa*-typing, identification of *S. aureus* virulence genes, and the prediction of AMR phenotype can be achieved directly from patient soft tissue biopsies using the present nanopore shotgun metagenomic sequencing protocol. However, MLST needs further optimization.

*spa*-typing and MLST are common genotypic screening methods for characterising *S. aureus* strains and are useful tools for detecting outbreaks and local epidemiology [8,22]. A culture-independent rapid genotyping protocol could help to quickly identify and contain local outbreaks, thus minimizing the potential consequences of such. In the present context, genotyping also serves as a quality check of sorts, assuring that the *S. aureus* isolates cultured during routine diagnostic were representative of the *S. aureus* clones of the respective patient’s OIAI. *spa*-typing, virulence-typing, and AMR gene detection results support this. MLST, despite the basecalling issues discussed below, also seems to support this when krocus and CGE results are considered together.

*spa*-typing results matched 100% between methods. However, metagenomic and WGS MLST analysed using CGE were suboptimal; CGE classification matched unambiguously in one patient’s samples (ID 140), uncertainly in one (ID 128), and incorrectly in the remaining four patients’ samples. It should be noted that the differences in these five MLST discrepancies were sequence types that varied by a single nucleotide in four cases (IDs 114, 128, 139, 140) and two nucleotides in the fifth (ID 114). Only a single A deletion separates ST22 and ST957 (ID 114), ST ST30 and ST4618 (IDs 139, 140), and ST6325 from a 100% match (128). In ST15 and ST5510 (ID 111), the difference was an *arcC* A deletion and an *yqil* C > T substitution. Indicating that the five metagenomic inconsistencies with Sanger sequencing were by a small margin of error.

Metagenomic krocus results matched Sanger fully in four of the six patient’s samples. As shown in Table 1 however, sequence discrepancies made certain typing of MLST difficult in cases where putative basecalling errors led either to incorrect alleles being assigned (ID 140) or to imperfect allele matches (ID 128).

Nanopore sequencing is known to have some issues resolving homopolymeric regions [23]. Despite polishing the metaflye assemblies with medaka, these issues persisted. Nanopore sequencing’s increased potential for erroneous basecalling in these areas at the *arcC* loci seems the cause for the present *arcC* discrepancies. ONT’s newly released R10 flow cell promises improved resolution of homopolymeric regions, warranting eventual re-testing of this portion of the protocol when the R10 becomes more widely available.

The analysis of raw nanopore reads using krocus, which types and displays each uncorrected read individually in the output file, yielded more easily interpreted MLST results than the CGE analysis of the metaflye assembled and medaka polished contigs. Sequencing error was still somewhat of an issue in the krocus results of two patients’ samples though (IDs 128, 140). It should be noted that krocus is dependent upon a manually updated local database. BioNumerics is updated automatically upon opening the software, and CGE synchs with pubMLST automatically.

Virulence genes detection matched 100% between nanopore shotgun metagenomic and isolate WGS. Many potential benefits of rapid identification virulence, genotype and resistance are conceivable. Identification of *S. aureus* virulence factors can give the clinician insight into an infection’s pathogenesis, and, as specific anti-virulence treatments and vaccines emerge, can inform the clinician’s choice of therapy [24,25]. Less cytotoxic strains of *S. aureus*, for example, have been shown to more easily evade host immunity [11]. Early identification of such strains might lead to better informed treatment decisions, helping to prevent chronic *S. aureus* infections. In addition, virulence factors such as Panton-Valentine Leukocidin (PVL) can also function as biomarkers in pathotyping strains [22].

The potential benefits of rapid AMR identification are significant. Expedient and correct OIAI antimicrobial treatment can potentially lead to reduced selection for antimicrobial resistant bacterial clones, fewer revision surgeries and device removals, increased functionality, and decreased postoperative mortality. The culture-independent metagenomic protocol’s ResFinder results predicted culture-based AST resistance phenotype in all six cases. Use of this protocol could amount to a significant reduction in our current 3.5-day median time to culture-based AST results [12].

Of the samples tested here, 33,087 (ID 128) was the minimum read amount required for the protocol. Each sequencing run was set to a duration of 48 h; however, 33,002 of the *S. aureus* reads in this run were produced within the first 24 h of sequencing. Sequencing hours 24–48 produced only an additional 85 reads. The speed at which each the other five nanopore runs were able to produce 33,087 *S. aureus* reads varied from about one hour to 12 h, relative to the varying concentrations of *S. aureus* and proportions of hDNA in each sample. Nanopore sequencing allows the user to monitor the sequencing results in near real-time, and to stop the run when enough data have been accrued. The additional time required of the genotyping, virulence and resistance gene detection programs used here is negligible, adding mere minutes to the total time. Had sample ID 128 been stopped after 24 h of sequencing, the total time to results for the samples analysed in the present study would have ranged from about nine to 30 h from biopsy.

AMR gene detection of the metagenomic sequencing of these samples was carried out in earlier work using ONT’s EPI2ME ARMA workflow [5]. All ARMA AMR results included genes that were not represented in the respective observed AST phenotypes. The genes included *tet38*, *sav1866*, *mepA*, *mepR*, *mgrA*, *arlS*, and *arlR* (Supplementary data, Table S4). These genes are reportedly present in over 99% of *S. aureus* genomes (The Comprehensive Antibiotic Resistance Database; https://card.mcmaster.ca/). They are associated with bacterial efflux systems and have the ability to confer resistance to several other antimicrobials when being expressed; however, expression is regulated by genetic variation, specific regulators, and conditions [26,27,28,29]. Their presence alone does not ensure the presence of AMR. It is, therefore, not unusual that the respective phenotypes were not reflected during AST. The presence of *vgaA* in one patient’s metagenomic data (ID 140) was due most likely to the *S. epidermidis* reads [30]. From the limited data analysed here, it would seem that the AMR gene detection results of CGE’s ResFinder provides a clearer picture of the *S. aureus* AMR phenotype. In addition to AMR gene detection, ResFinder results provide a translation of genotypes into predicted phenotypes. However, as of this writing, ResFinder has only been validated for 13 bacterial species/genera of major public health relevance (*S. aureus* included), and prediction of AMR phenotype from species other than those six may require more extensive knowledge of AMR [16].

Nanopore sequencing has been successfully employed by others for outbreak analysis [31], WGS *spa*-typing and virulence typing [32] and targeted MLST [33] of *S. aureus*. These analyses were performed on isolates, however.

The study’s limitations lie primarily in the small sample group that contained “sufficient” *S. aureus* sequencing reads. Additionally, as this is a single centre study in a geographic location with a low prevalence of AMR, the antimicrobial resistance detection results might translate differently to areas with a greater pathogen diversity and prevalence of AMR. Norway is a country with a relatively low prevalence of methicillin resistant *S. aureus* (MRSA). No MRSA were among the samples included in the present study. In the current report, the Norwegian program for the surveillance of antimicrobial resistance (NORM) reports that MRSA comprised no more than 1.3% of the *S. aureus* isolated tested from human sources [34]. More diverse AMR among the *S. aureus* strains might have given better insight into the protocol’s ability to predict AMR phenotype.

The metagenomic read threshold for the feasibility of the present protocol apparently lies roughly between 10,000 and 33,000 *S. aureus* reads and a median depth of coverage somewhere between 11 and 33. When aligned to the isolate assemblies, the metagenomic assemblies’ median breadth of coverage of the isolates’ *S. aureus* chromosome was 95% [69.2%–99.9%], and pair-wise identity matched a median of 99.88%. In two patients’ samples’ isolates’ assemblies, plasmids were observed. Plasmid coverages were 50% and 14.3%. Assembly breadth of coverage was not proportional to amount of metagenomic nanopore reads, but rather an apparent partiality in the PCR amplification of certain *S. aureus* genomic fragments during pre-sequencing sample preparation. This is somewhat troubling in that it implies that the genes necessary for *spa*-typing, MLST, and virulence and AMR gene detection might not have been present for downstream analyses had they been carried on these missing areas. This was not an issue in the current work.

## 5. Conclusions

These results indicate that *S. aureus spa*-typing, and identification of virulence and antimicrobial resistance genes are possible using nanopore shotgun metagenomics sequencing directly on tissue biopsies analysed with the protocol presented here. Culture-free detection of virulence genes and prediction of AMR phenotype can contribute to more timely and more accurately targeted treatment of OIAI. The protocol requires optimization before it can be reliably used for MLST classification of *S. aureus*. Apart from *spa*-typing, specific to *S. aureus*, the protocol has the potential to be used in the analysis of other species and types of infections.

## Figures and Tables

**Table 1:** Overview of the *S. aureus* MLST results across the different protocols.

Patient	Sanger BioNumerics Isolate	Nanopore CGE MLST Isolate	Nanopore CGE MLST Metagenomic	Nanopore Krocus Isolate	Nanopore Krocus Metagenomic
ID 111	15	5510? * *yqil* 99.8%	5510? * *yqil* 99.8%	15	15
ID 114	22	22	957	22	22
ID 128	6325	6325? * *arcC* 99.8%	6325? * *arcC* 99.8%	6325? * *arcC* 99.8%	Not found *glpF* 99.9%
ID 139	30	4618	4618	30	30
ID 140	6326	6326? * *arcC*, *gmk*, *pta*, *tpi* 99.8%	6326	6326? * *arcC*, *gmk*, *pta*, *tpi* 99.3%	6326 or 582
ID 141	30	4618	4618	30 * *aroE* 99.9%	30

* Percent similarity shown where profiles were less than 100% match.

## Data Availability

The *S. aureus* sequencing data have been deposited in the National Center for Biotechnology Information (NCBI) under bioproject ID PRJNA714681. Additional data provided upon request. Requests can be directed to the corresponding author.

## Supplementary Materials

The following are available online at https://www.mdpi.com/article/10.3390/microorganisms9040707/s1. Figure S1. Alignment of the assembled metagenomic assemblies with each’s respective isolate. Table S1. Nanopore sequencing QC metrics. Table S2. metaflye assembly metrics. Table S3. VirulenceFinder results. Table S4. Results of resistance phenotype (AST) and resistance genes detected.
