# Supplementary material for: Culture-Independent Genotyping, Virulence and Antimicrobial Resistance Gene Identification of Staphylococcus aureus from Orthopaedic Implant-Associated Infections
Source: Microorganisms. 2021 Mar 30;9(4):707. doi: 10.3390/microorganisms9040707 (PMC8065434; doi:10.3390/microorganisms9040707)
Supplement: Supplementary file 1 [file microorganisms-09-00707-s001.pdf]

Supplementary Material

Culture-independent Genotyping, Virulence and Antimicrobial Resistance Gene Identification of *Staphylococcus aureus* from Orthopaedic Implant-associated Infections

J. Christopher Noone [1,2,*], Fabienne Antunes Ferreira [1,3] and Hege Vangstein Aamot [1,4]

[1]Department of Microbiology and Infection Control, Akershus University Hospital, 1478 Lørenskog, Norway;

[2]Faculty of Medicine, University of Oslo, 0316 Oslo, Norway

[3]Departamento de Microbiologia, Imunologia e Parasitologia, Universidade Federal de Santa Catarina (UFSC), Florianópolis, Brazil. Postcode 88040-900

[4]Department of Clinical Molecular Biology (EpiGen), Akershus University Hospital and University of Oslo, 1478 Lørenskog, Norway

[*]Correspondence: cnoo@ahus.no

Contents

Table S1. Nanopore sequencing QC metrics

Table S2. metaflye assembly metrics

Figure S1. Alignment of the assembled metagenomic assemblies with each's respective isolate

Table S3. VirulenceFinder results

Table S4. Results of resistance phenotype (AST) and resistance genes detected.

Table S1. Nanopore sequencing QC metrics, totals for each patient: meta* (shotgun metagenomic sequencing data), iso* (isolate WGS data).

Patient ID	Total reads	Total bases	Mean quality score	Mean read length (bp)
**iso111**	293910	1.9 Gb	13.55	6619
**meta111**	394935	1.1 Gb	12.46	2779
**iso114**	76076	186.8 Mb	12.21	2456
**meta114**	1740658	124.6 Mb	12.54	2811
**iso128**	137345	405.6 Mb	12.06	2963
**meta128**	37382	111.4 Mb	12.38	2980
**iso139**	205789	651.7 Mb	12.45	3166
**meta139**	41736	139.9 Mb	12.54	3351
**iso140**	53764	70.3 Mb	11.25	1307
**meta140**	2273296	9.8 Gb	12.87	4324
**iso141**	275640	1.2 Gb	12.58	4272
**meta141**	2744512	11.9 Gb	12.89	4335

Table S2. metaflye assembly metrics: meta* (shotgun metagenomic sequencing data), iso* (isolate WGS data).

Patient ID	Contig	Length	Depth of coverage
**meta111**			
	8	953233	272
	11	895294	225
	6	436786	232
	5	122429	233
	3	120684	247
	10	111219	238
	9	77748	260
	7	55734	155
	12	27619	48
	13	20696	1001
	2	742	10
**meta114**			
	2	1030116	995
	1	840535	945
	3	572122	961
	6	202434	912

	5	112560	884
	7	748	989
**meta128**			
	19	468070	38
	18	341601	30
	34	186963	30
	30	176411	29
	33	174906	35
	3	130882	37
	25	120045	36
	1	111175	35
	28	83346	28
	32	79091	28
	9	74636	31
	17	68801	29
	21	68765	30
	29	61305	29
	6	56539	31
	20	52617	31
	22	52172	42
	31	50487	32

10	50329	32
16	48335	32
26	46236	39
27	43935	37
24	43016	32
35	38494	23
12	37503	30
11	33773	30
8	23277	25
5	21162	28
13	19307	24
7	17855	28
14	15465	21
15	13732	25
2	8036	25

meta139

12	1152988	48
9	976982	34
11	266425	35
8	193349	40
10	177700	39

	14	43902	41
	3	5297	40
	6	2903	45
	4	2722	66
	7	1837	44
	1	1259	23
	5	870	204
	2	837	16
**meta140**			
	4	1293595	969
	3	824156	975
	5	618099	966
**meta141**			
	12	692856	408
	11	435165	271
	3	254053	287
	13	236318	345
	16	219621	281
	17	198927	310
	18	195804	291
	6	191249	272

	15	124339	302
	2	93434	327
	7	64203	283
	5	41675	360
	9	36400	302
	1	32786	365
	8	25817	356
	19	7124	168
	4	7082	254
	21	4017	293
	20	2720	236
	10	2393	367
**iso111**			
	1	2803888	995
	2	41435	646
**iso114**			
	1	2770789	64
	2	3117	757
**iso128**			
	1	2823596	142
**iso139**			

	1	2816259	231
**iso140**			
	1	2736340	22
**iso141**			
	1	2883453	402

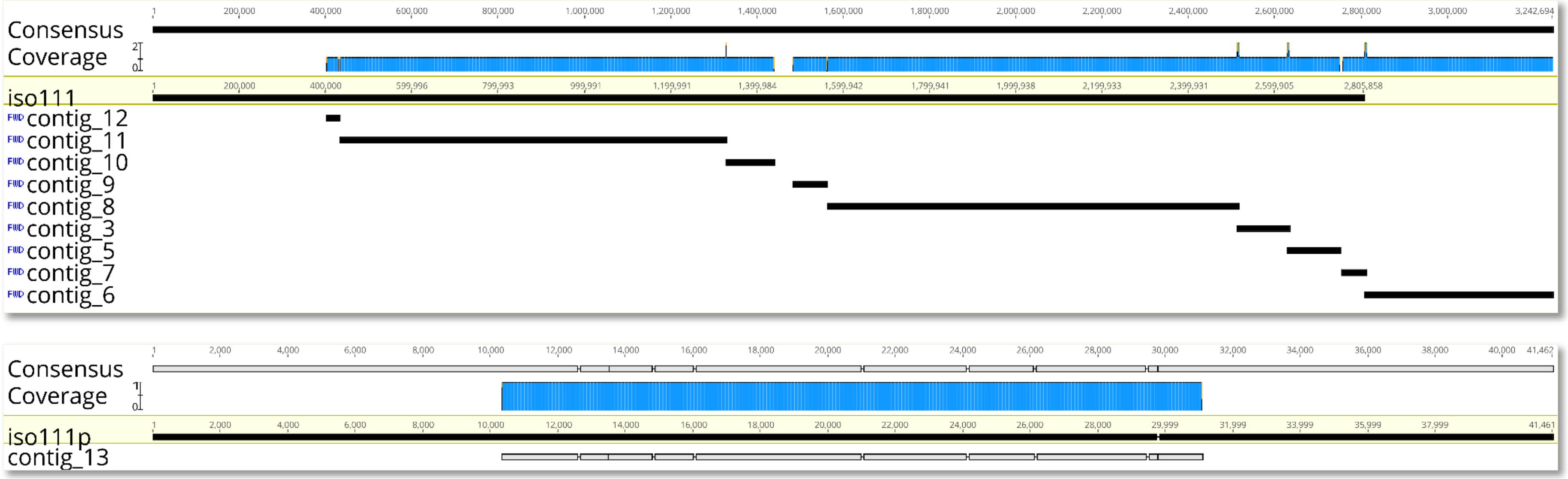

Consensus
Coverage
iso111
contig_12
contig_11
contig_10
contig_9
contig_8
contig_3
contig_5
contig_7
contig_6
1
200,000
400,000
600,000
800,000
1,000,000
1,200,000
1,400,000
1,600,000
1,800,000
2,000,000
2,200,000
2,400,000
2,600,000
2,800,000
3,000,000
3,242,694
599,996
799,993
999,991
1,199,991
1,399,984
1,599,942
1,799,941
1,999,938
2,199,933
2,399,931
2,599,905
2,805,858
Consensus
Coverage
iso111p
contig_13
2,000
4,000
6,000
8,000
10,000
12,000
14,000
16,000
18,000
20,000
22,000
24,000
26,000
28,000
30,000
32,000
34,000
36,000
38,000
40,000
41,462
29,999
31,999
33,999
35,999
37,999
41,461

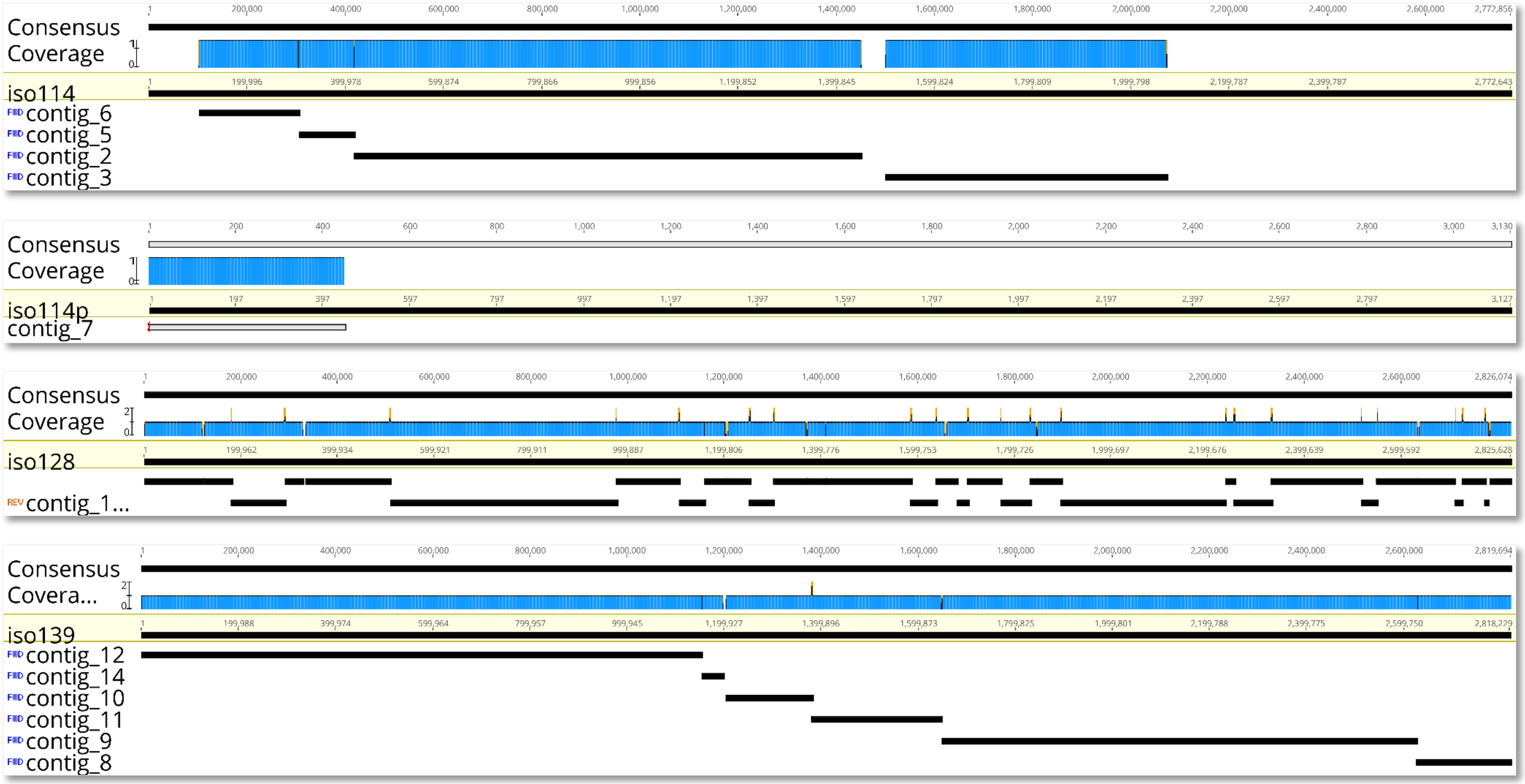
Consensus
Coverage
iso114
contig_6
contig_5
contig_2
contig_3
Consensus
Coverage
iso114p
contig_7
Consensus
Coverage
iso128
REV contig_1...
Consensus
Covera...
iso139
contig_12
contig_14
contig_10
contig_11
contig_9
contig_8

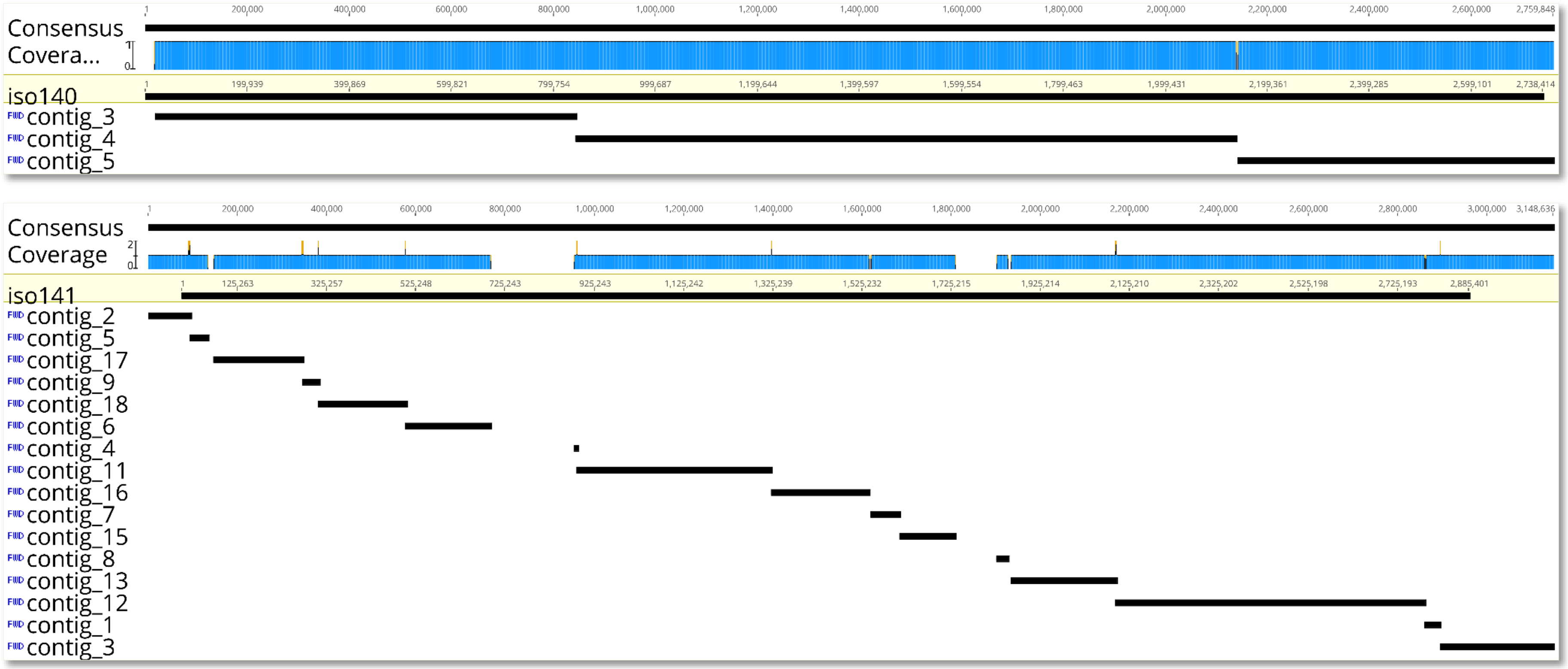

Figure S1. Alignment of the assembled metagenomic assemblies with each's respective isolate. The assembled metagenomic contigs are shown here aligned to each respective isolate's chromosome (iso*). Isolate nanopore WGS assemblies (pale yellow bar) were set as the reference genome for each patient's respective metagenomic assembly (contig*). Two isolates revealed the presence of plasmids (IDs iso111p, iso114p). The blue bar reflects the area of the isolate genome covered by the metagenomic

contigs. The alignment of ID 128 has been collapsed for easier viewing. Metagenomic contigs overhanging the ends of the isolate reference are due to the circular genome of *S. aureus*. Figure rendered in Geneious v.2021.0.3.

Table S3. VirulenceFinder results. *S. aureus* virulence genes detected in shotgun metagenomic sequencing data (meta*) and isolate WGS data (iso*) (https://cge.cbs.dtu.dk/services/VirulenceFinder/).

Patient 111		Patient 114		Patient 128		Patient 139		Patient 140		Patient 141	
**iso111**	**meta111**	**iso114**	**meta114**	**iso128**	**meta128**	**iso139**	**meta139**	**iso140**	**meta140**	**iso141**	**meta141**
aur	*Aur*	*aur*	*aur*	*aur*	*aur*	*aur*	*aur*	*aur*	*aur*	*aur*	*aur*
								eta	*eta*		
hlgA	*hlgA*	*hlgA*	*hlgA*	*hlgA*	*hlgA*	*hlgA*	*hlgA*	*hlgA*	*hlgA*	*hlgA*	*hlgA*
hlgB	*hlgB*	*hlgB*	*hlgB*	*hlgB*	*hlgB*	*hlgB*	*hlgB*	*hlgB*	*hlgB*	*hlgB*	*hlgB*
hlgC	*hlgC*	*hlgC*	*hlgC*	*hlgC*	*hlgC*	*hlgC*	*hlgC*	*hlgC*	*hlgC*	*hlgC*	*hlgC*
lukD	*lukD*							*lukD*	*lukD*		
lukE	*lukE*							*lukE*	*lukE*		
		sak	*sak*	*sak*	*sak*	*sak*	*sak*			*sak*	*sak*
scn	*scn*	*scn*	*scn*	*scn*	*scn*	*scn*	*scn*	*scn*	*scn*	*scn*	*scn*
										sea	*sea*
		seg	*seg*	*seg*	*seg*	*seg*	*seg*			*seg*	*seg*
		sei	*sei*	*sei*	*sei*	*sei*	*sei*			*sei*	*sei*
		sem	*sem*	*sem*	*sem*	*sem*	*sem*			*sem*	*sem*
		sen	*sen*	*sen*	*sen*	*sen*	*sen*			*sen*	*sen*
		seo	*seo*	*seo*	*seo*	*seo*	*seo*			*seo*	*seo*
		seu	*seu*	*seu*	*seu*	*seu*	*seu*			*seu*	*seu*
splA	*splA*							*splA*	*splA*		
splB	*splB*							*splB*	*splB*		
splE	*splE*			*splE*	*splE*	*splE*	*splE*	*splE*	*splE*	*splE*	*splE*
		tst	*tst*	*tst*	*tst*	*tst*	*tst*			*tst*	*tst*

Table S4. Results of resistance phenotype (AST) and resistance genes detected. Abbreviations are as follows: AST (antimicrobial susceptibility testing), ResFinder (https://cge.cbs.dtu.dk/services/ResFinder/), *iso (data from WGS *S. aureus* isolate sequencing), *meta (data from shotgun metagenomic sequencing), R (resistant), I (susceptible, increased exposure), S (susceptible).

Patient ID	AST (phenotype)	ResFinder_iso (% similarity)	ResFinder_meta (% similarity)	EPI2ME_iso (% similarity)	EPI2ME_meta (% similarity)
**111**	**Find: *S. aureus***<br>Penicillin (R)<br>Ciprofloxacin (S)<br>Erythromycin (S)<br>Fusidic acid (S)<br>Gentamicin (S)<br>Clindamycin (S)<br>Chloramphenicol (S)<br>Linezolid (S)<br>Oxacillin (S)<br>Rifampicin (S)<br>Tetracycline (S)<br>Trimethoprim/sulfamethoxazole (S)	*blaZ* (100)	*blaZ* (100)	*blaZ* (91.9)<br>*tet38* (94)<br>*sav1886* (93.6)<br>*mepA* (93.4)<br>*mgrA* (94.1)<br>*arlS* (94)<br>*mepR* (94.2)<br>*arlR* (93.8)	*blaZ* (92)<br>*tet38* (93.6)<br>*sav1886* (93.3)<br>*mepA* (93.9)<br>*mgrA* (94.3)<br>*arlS* (94)<br>*mepR* (94.9)

**114**	**Find:** ***S. aureus***<br>Penicillin (R)<br>Ciprofloxacin (S)<br>Erythromycin (S)<br>Fusidic acid (S)<br>Gentamicin (S)<br>Clindamycin (S)<br>Linezolid (S)<br>Oxacillin (S)<br>Rifampicin (S)<br>Tetracycline (S)<br>Trimethoprim/sulfamethoxazole (S)	*blaZ* (100)	*blaZ* (99.89)	*blaZ* (91.5)<br>*tet38* (93.8)<br>*sav1886* (93.1)<br>*mgrA* (94.5)<br>*arlS* (93.2)<br>*arlR* (93.2)<br>*mepR* (93.3)<br>*mepA* (93.8)	*blaZ* (91)<br>*tet38* (93.5)<br>*sav1866* (93.4)<br>*mgrA* (94)<br>*arlS* (93.5)<br>*arlR* (93.7)<br>*mepR* (93)
**128**	**Find:** ***S. aureus***<br>Penicillin (R)<br>Tetracycline (R)<br>Ciprofloxacin (I)<br>Erythromycin (S)<br>Fusidic acid (S)<br>Gentamicin (S)<br>Clindamycin (S)<br>Linezolid (S)<br>Oxacillin (S)<br>Rifampicin (S)<br>Trimethoprim/sulfamethoxazole (S)	*blaZ* (100)<br>*tetK* (100)	*blaZ* (99.88)<br>*tetK* (100)	*blaZ* (91.2)<br>*tetK* (93.9)<br>*sav1866* (93.7)<br>*tet38* (92.9)<br>*mepR* (94.3)<br>*mgrA* (94)<br>*mepA* (92.5)<br>*arlS* (92.6)<br>*arlR* (93.1)	*blaZ* (89.5)<br>*tetK* (93.5)<br>*sav1866* (93.1)<br>*tet38* (92.4)<br>*mepR* (93.5)<br>*mgrA* (93.8)<br>*mepA* (92.2)<br>*arlS* (92.1)<br>*arlR* (93.4)

**139**	**Find:** ***S. aureus***<br>Penicillin (R)<br>Ciprofloxacin (S)<br>Erythromycin (S)<br>Fusidic acid (S)<br>Gentamicin (S)<br>Clindamycin (S)<br>Linezolid (S)<br>Oxacillin (S)<br>Rifampicin (S)<br>Tetracycline (S)<br>Trimethoprim/sulfamethoxazole (S)	*blaZ* (100)	*blaZ* (99.88)	*blaZ* (91.2)<br>*tet38* (93.2)<br>*mepA* (92.7)<br>*sav1866* (93.6)<br>*arlS* (93.2)<br>*mepR*(93.2)<br>*arlR* (93.7)<br>*mgrA* (94.2)	*blaZ* (91.3)<br>*tet38* (93.2)<br>*mepA* (92.6)<br>*sav1866* (92.9)<br>*arlS* (93)<br>*mepR*(96.2)<br>*arlR* (94.4)
**140**	**Find:** ***S. aureus***<br>Penicillin (R)<br>Ciprofloxacin (S)<br>Erythromycin (S)<br>Fusidic acid (S)<br>Gentamicin (S)<br>Clindamycin (S)<br>Linezolid (S)<br>Oxacillin (S)<br>Rifampicin (S)<br>Tetracycline (S)<br>Trimethoprim/sulfamethoxazole (S)	*blaZ* (97.55)	*blaZ* (100)	*blaZ* (90.6)<br>*tet38* (94.0)<br>*sav1866* (94)<br>*mepA* (95)<br>*mgrA* (93.8)<br>*arlS* (94.2)<br>*arlR* (93.9)<br>*mepR* (91.6)	*blaZ* (91.2)<br>*tet38* (94.1)<br>*sav1866* (93.7)<br>*mepA* (93.5)<br>*mgrA* (94.4)<br>*arlS* (94.2)<br>*arlR* (93.4)<br>*mepR* (94.8)<br>*vgaA* (91.9)

**141**	**Find:** ***S. aureus***<br>Penicillin (R)<br>Ciprofloxacin (S)<br>Erythromycin (S)<br>Fusidic acid (S)<br>Gentamicin (S)<br>Clindamycin (S)<br>Linezolid (S)<br>Oxacillin (S)<br>Rifampicin (S)<br>Tetracycline (S)<br>Trimethoprim/sulfamethoxazole (S)	*blaZ* (100)	*blaZ* (100)	*blaZ* (91.8)<br>*tet38* (93.1)<br>*sav1866* (93.3)<br>*mepA* (93.1)<br>*mgrA* (94.3)<br>*arlS* (93)<br>*arlR* (93.2)<br>*mepR* (93.5)	*blaZ* (91.5)<br>*tet38* (92.9)<br>*sav1866* (93.2)<br>*mepA* (92.6)<br>*mgrA* (94.1)<br>*arlS* (92.9)<br>*arlR* (93.4)<br>*mepR* (93.6)
